# Predictive factors for the benefit of triple‐drug transarterial chemoembolization for patients with unresectable hepatocellular carcinoma

**DOI:** 10.1002/cam4.2355

**Published:** 2019-06-17

**Authors:** MinKe He, Qing Li, JingXian Shen, GuoSheng Tan, QiJiong Li, JiaYing Lai, Wei Wei, YaoJun Zhang, RuHai Zou, MinShan Chen, RongPing Guo, Ming Shi

**Affiliations:** ^1^ Department of Hepatobiliary Oncology Sun Yat‐sen University Cancer Center, State Key Laboratory of Oncology in South China, Collaborative Innovation Center for Cancer Medicine Guangzhou China; ^2^ Department of Ultrasonography Sun Yat‐sen University Cancer Center, State Key Laboratory of Oncology in South China, Collaborative Innovation Center for Cancer Medicine Guangzhou China; ^3^ Department of Radiology Sun Yat‐sen University Cancer Center, State Key Laboratory of Oncology in South China, Collaborative Innovation Center for Cancer Medicine Guangzhou China; ^4^ Department of Interventional Radiology The First Affiliated Hospital of Sun Yat‐sen University Guangzhou China; ^5^ HuiDong Senior Middle School Huidong China

**Keywords:** overall survival, predictive factor, propensity score matching, single‐drug chemotherapy, transarterial chemoembolization, triple‐drug chemotherapy

## Abstract

**Background:**

Compared with single‐drug TACE, our previous phase III study demonstrated that triple‐drug transarterial chemoembolization (TACE) prolonged overall survival (OS) in patients with unresectable hepatocellular carcinoma (HCC). The aim of this study was to find which patients can benefit from the triple drugs TACE compared with single‐drug TACE.

**Methods:**

Patients in the triple‐drug TACE arm received sponge embolization and emulsions composed of 50 mg epirubicin, 50 mg lobaplatin, 6 mg mitomycin C, and lipiodol, while patients in the single‐drug TACE arm received sponge embolization and emulsions composed of 50 mg epirubicin and lipiodol. From July 2007 to November 2009, 244 patients (224 men and 20 women; age ranged from 21 to 75 years) from our phase III study formed the initial cohort. From January 2010 to June 2015, external validation cohort was composed of 449 patients (411 men and 38 women; age ranged from 18 to 75 years) from another institution. The validation cohort after propensity score matching (PSM) (n = 374) was analyzed. Cox proportional hazard model was used to evaluate the interaction term between treatments for each subgroup. This retrospective study was approved by the institutional review board at each center.

**Results:**

No difference was observed in the baseline characteristic of three cohorts. This exploratory analysis showed that triple‐drug TACE brought a survival benefit in the initial cohort, validation cohort (before PSM), and validation cohort (after PSM) compared with single‐drug TACE. The outcomes of three cohorts all showed that a significantly greater OS triple‐drug chemotherapy benefit versus single‐drug chemotherapy was seen in patients with large tumors (larger than 10 cm) while no survival difference was seen in patients with small tumors (10 cm or smaller).

**Conclusions:**

Triple‐drug TACE seems to benefit patients with HCC larger than 10 cm in particular compared with single‐drug TACE.

## 1 INTRODUCTION

Transarterial chemoembolization (TACE) is the first‐line treatment for patients with unresectable hepatocellular carcinoma (HCC) at intermediate stage, and nexavar (sorafenib) is the first‐line treatment for patients with HCC at advanced stage.^1^, ^2^ According to Barcelona Clinic Liver Cancer (BCLC) staging system, intermediate stage (BCLC B) is formed by those patients with single large HCCs and those with multifocal disease who are asymptomatic and do not present vascular invasion or extrahepatic spread, and advanced stage (BCLC C) is formed by those patients with symptoms and/or present vascular invasion or extrahepatic spread.^3^ Different from Western guidelines, Asian guidelines recommend TACE for advanced HCC.^4^, ^5^ Additionally, a randomized, phase III of advanced HCC showed that TACE plus sorafenib had noninferiority in overall survival (OS) (12.8 vs 10.8 months, *P* = 0.29) but superiority in time to progression (5.3 vs 3.5 months, *P* = 0.003), progression‐free survival (5.2 vs 3.6 months, *P* = 0.01) and response rate (60.6% vs 47.3%, *P* = 0.005) compared with sorafenib monotherapy, and TACE plus sorafenib had longer survival rate in patients who received ≥2 TACE sessions (18.6 vs 10.8 months, *P* = 0.0055) in the subgroup analysis.^6^

Conventional TACE is composed of chemotherapy and embolization. However, which one played the leading role was controversial. Some studies showed that chemotherapy made a positive effect, and adding embolization did not improve survival.^7^, ^8^, ^9^, ^10^ In contrast, others believed that embolization was important, and chemotherapy was useless except adding adverse events.^11^, ^12^, ^13^ Additionally, a meta‐analysis including six randomized controlled trials demonstrated that there was no superiority between TACE and bland embolization in HCC.^14^

However, our multicenter, randomized, phase III trial including HCC at intermediate or advanced stage showed that triple‐drug chemolipiodolization with gelatin‐sponge embolization improved OS compared single‐drug chemolipiodolization with gelatin‐sponge embolization (10.5 vs 5.9 months; hazard ratio [HR], 1.8; 95% confidence interval [CI], 1.35‐2.39; *P* = 0.0003). While there was no OS benefit of TACE with gelatin‐sponge embolization versus TACE without gelatin‐sponge embolization (10.5 vs 10.1 months; HR, 1.21; 95% CI, 0.91‐1.61; *P* = 0.2).^15^ In addition, no statistically significant difference in the incidence of adverse event or serious adverse event was observed between triple‐drug TACE and single‐drug TACE.

Although there are a number of clinical scoring systems that help assess patient prognosis after TACE,^16^, ^17^, ^18^ predictive factors for the additional benefit of triple‐drug chemotherapy compared with single chemotherapy are lacking. A predictive factor, which is a parameter used to distinguish subgroup of patients who can benefit most from a specific treatment, is different from a prognostic factor, which is a baseline characteristics related to the natural processes of the disease in spite of treatment.^19^ The data of prospective randomized trials are ideal to research predictive factors. The purpose of this study was to find out baseline parameters that predicted the survival benefit of triple‐drug chemotherapy on OS compared with single‐drug TACE in patients with unresectable HCC at intermediate or advanced stage.

## 2 METHODS

### 2.1 Study design

This prior article (ClinicalTrials.gov number NCT00493402) dealt with the development of the important role of triple‐drug chemotherapy in the TACE^15^ whereas in this manuscript, we reported on which patients can benefit from triple‐drug TACE in particular. In this retrospective analysis, from July 2007 to November 2009, 244 patients who have been previously reported in our phase III clinical study^15^ formed the initial cohort. Besides, between January 2010 and June 2015, 449 patients who met the following criteria and received the same treatment as our phase III study therapy at the First Affiliated Hospital of Sun Yat‐Sen University formed the external validation cohort. Criteria of patients enrolled in the external validation cohort were the same as the phase III clinical study: (a) age ranging from 18 to 75 years; (b) the size of main tumor larger than 7 cm; (c) Child‐Pugh A liver cirrhosis and adequate organ function (platelet count greater than 60000/μL; hemoglobin greater than 8.5 g/dL; and prothrombin time less than 3 seconds above control; albumin greater than 3.5 g/dL; total bilirubin less than 1.5 mg/dL; and alanine aminotransferase and aspartate aminotransferase less than 5× upper limit of normal; serum creatinine less than 1.5 × upper limit of normal); (d) Eastern Cooperative Oncology Group performance status (ECOG PS) of 0 to 1; (e) unresectable HCC at intermediate or advanced stage; and (f) with no previous treatment. Exclusion criteria were as follows: (a) evidence of extrahepatic metastases; (b) cardiac ventricular arrhythmias requiring antiarrhythmic therapy; (c) evidence of hepatic decompensation including ascites, gastrointestinal bleeding or hepatic encephalopathy.

Even though we have demonstrated that triple‐drug TACE was superior to single‐drug TACE, no guideline recommended the triple‐drug regimen as the standard regimen. Therefore, some doctors used this triple‐drug regimen while others still used single‐drug regimen at the First Affiliated Hospital of Sun Yat‐sen University. This study procedure was approved by the ethics committee of the Sun Yat‐sen University Cancer Center and the First Affiliated Hospital of Sun Yat‐sen University, and was performed in accordance with the Declaration of Helsinki. Each patient included in the study signed informed consent.

### 2.2 TACE procedure

Both the initial cohort and validation cohort were conducted on the basis of our previously reported protocol.^15^ Patients in the triple‐drug TACE arm (triple arm) received emulsions composed of 50 mg epirubicin (H20000497; Pharmorubicin, Pfizer, Wuxi, Jiangsu, China), 50 mg lobaplatin (H20080359; Hainan Changan International Pharmaceutical Co. Ltd., Haikou, Hainan, China), 6 mg mitomycin C (H33020786; Zhejiang Hisun Pharmaceutical Co. Ltd., Taizhou, Zhejiang, China), and lipiodol (H20150099; Lipiodol UltraFluide; Guerbet Laboratories, Aulnay Sous Bois, Paris, France). The injection was stopped when the point of near stasis within the feeding artery occured. Then gelatin‐sponge particles (H32024096; Gelfoam; Hanzhou alc Ltd, China) were used to achieve embolization of the tumor‐feeding artery. Patients in the single‐drug TACE arm (single arm) were treated with 50 mg epirubicin, lipiodol and gelatin‐sponge particles as above, but without mitomycin C and lobaplatin.

Patients who received TACE with single‐drug regimen or TACE with triple‐drug regimen at the First Affiliated Hospital of Sun Yat‐Sen University were divided into the single‐drug TACE arm or the triple‐drug TACE arm, respectively. Patients who received only one kind of TACE were included in analysis, and patients who received treatment crossover from TACE with single‐drug regimen to TACE with triple‐drug regimen or from TACE with triple‐drug regimen to TACE with single‐drug regimen were excluded.

All patients with HBV received antiviral therapy. Subsequent TACE was performed as the same as initial TACE and on an “on‐demand” as follows: the presence of active lesions, and adequate liver function [Child‐Pugh score 5‐6]). The initial cohort used the original OS date. The validation cohort was censored on 31 December 2016.

### 2.3 Statistical analysis

The two tailed chi‐squared test for categorical variables were used to compare baseline characteristics. The Kaplan‐Meier method was used to calculate survival curves, and the log‐rank test was used for univariate analysis. A multivariate Cox analysis included all factors irrespective of *P* value in the univariate analysis. Enter method was used in cox regression (Enter all variables in the model in one single step, without checking). The significance level of 0.05 was used to confirm statistical significance. The Statistical Package used to perform analysis was SAS software (version 9.0; SAS, Cary, NC).

Retrospective variables between groups in the validation cohort might have adverse impact on the outcomes. Therefore, propensity score matching (PSM) analysis was conducted to reduce the influence of selection bias and potential confounding factors between arms, and the data after PSM formed the validation cohort (after PSM).^20^, ^21^ All parameters were included in PSM (age, sex, neutrophil:lymphocyte ratio [NLR], prothrombin time, hepatitis B surface antigen, alanine aminotransferase, aspartate aminotransferase, alkaline phosphatase, glutamyl transpeptidase [GGT], albumin (ALB), total bilirubin (TBil), alpha‐fetoprotein (AFP), tumor size, tumor number, and portal vein tumor thrombus [PVTT]). Matched pairs were then formed using a one‐to‐one nearest‐neighbor caliper of width 0.2. The purpose of this exploratory analysis was to find out possible predictors for OS benefit from triple‐drug chemotherapy when compared with single chemotherapy. OS was measured from the date of initial TACE to death from any cause (or was censored at the time of data cutoff). The cutoff of continuous variables was consistent with our previous phase III study.^15^ To separately evaluate the predictive value of each parameter for predicting the survival benefit, each parameter's interaction term with treatment was tested in a model containing only the baseline parameter, the treatment and their interaction. The above statistical method was applied in the validation cohort.

## 3 RESULTS

### 3.1 Patient characteristics

In the initial cohort, 244 patients (122 patients in each arm) from our phase III study were analyzed, ranging from 21 to 75 years and including 224 men and 20 women. In the external validation cohort, 449 patients (226 patients in single arm and 223 patients in triple arm) were included in the study, ranging from 18 to 75 years and including 411 men and 38 women. After performing propensity score matching, we derived one‐to‐one paired cohorts (187 patients in each arm), ranging from 21 to 75 years and including 346 men and 28 women. No significantly difference was observed in the baseline characteristics of the initial cohort, validation cohort (before PSM), and validation cohort (after PSM) (Table 1). Most patients had HBV infection at baseline (93.4% in the initial cohort, 88.4% in the validation cohort [before PSM], and 90.6% in the validation cohort [after PSM]). The mean size of tumor was 11.5 cm, 10.6 cm, and 10.6 cm in the initial cohort, validation cohort (before PSM), and validation cohort (after PSM), respectively.

**Table 1:** Baseline patient and disease characteristics

	Initial cohort			External validation cohort			External validation cohort‐PSM		
	Triple arm (n = 122)	Single arm (n = 122)	*P*	Triple arm (n = 223)	Single arm (n = 226)	*P*	Triple arm (n = 187)	Single arm (n = 187)	*P*
Age, y			0.5996			0.0723			0.8354
≤50	77	72		129	111		104	101	
>50	45	50		94	115		83	86	
Sex			0.2428			0.0612			0.8446
Male	115	109		210	201		174	172	
Female	7	13		13	25		13	15	
NLR			0.8968			0.2924			0.4592
≤3	72	70		136	126		117	109	
>3	50	52		87	100		70	78	
PT, s			0.1331			0.0536			>0.9999
≤14	109	100		195	182		160	161	
>14	12	21		28	44		27	26	
HBsAg			>0.9999			0.3025			0.4781
Positive	114	114		201	196		172	167	
Negative	8	8		22	30		15	20	
ALT, U/L			>0.9999			0.6943			0.3859
≤40	40	41		81	78		70	61	
>40	82	81		142	148		117	126	
AST, U/L			>0.9999			0.2700			0.5076
≤45	24	23		45	36		38	32	
>45	98	99		178	190		149	155	
ALP, U/L			0.5091			0.0846			0.3469
≤110	49	43		100	83		85	75	
>110	73	79		123	143		102	112	
GGT, U/L			0.1806			0.3334			0.4791
≤100	35	25		63	54		52	45	
>100	87	97		160	172		135	142	

	Initial cohort			External validation cohort			External validation cohort‐PSM		
	Triple arm (n = 122)	Single arm (n = 122)	*P*	Triple arm (n = 223)	Single arm (n = 226)	*P*	Triple arm (n = 122)	Single arm (n = 122)	*P*
ALB, g/L			0.3033			0.1108			>0.9999
≤37	27	35		52	68		45	46	
>37	95	87		171	158		142	141	
TBil, µmol/L			0.6439			0.2563			>0.9999
≤20	97	93		179	171		152	152	
>20	25	29		44	55		35	35	
AFP, ng/mL			0.8929			0.3306			0.9158
≤200	43	41		79	91		73	75	
>200	79	81		144	135		114	112	
Tumor size, cm			0.4341			0.0895			>0.9999
≤10	46	53		101	121		90	91	
>10	76	69		122	105		97	96	
Tumor number			0.6971			0.7029			0.8346
Single	49	53		96	93		79	82	
Multiple	73	69		127	133		108	105	
PVTT			0.5911			0.9202			0.9129
Absence	82	77		151	152		125	123	
Presence	40	45		72	74		62	64	
BCLC stage			0.5911			>0.9999			>0.9999
Stage B	82	77		145	147		119	118	
Stage C	40	45		78	79		68	69	

Note

*P* values were calculated using a two‐sided Chi‐squared test

Abbreviations: AFP, alpha‐fetoprotein; ALB, albumin; ALP, alkaline phosphatase; ALT, alanine aminotransferase; AST, aspartate aminotransferase; BCLC, Barcelona Clinic Liver Cancer; GGT, glutamyl transpeptidase; HBsAg, hepatitis B surface antigen; NLR, neutrophil:lymphocyte ratio; PSM, propensity score matching; PT, prothrombin time; PVTT, portal vein tumor thrombus; Single arm, TACE with single‐drug chemotherapy; TACE, transarterial chemoembolization; TBil, total bilirubin; Triple arm, TACE with triple‐drug chemotherapy.

### 3.2 Prognostic factors for survival

**Figure 1:**
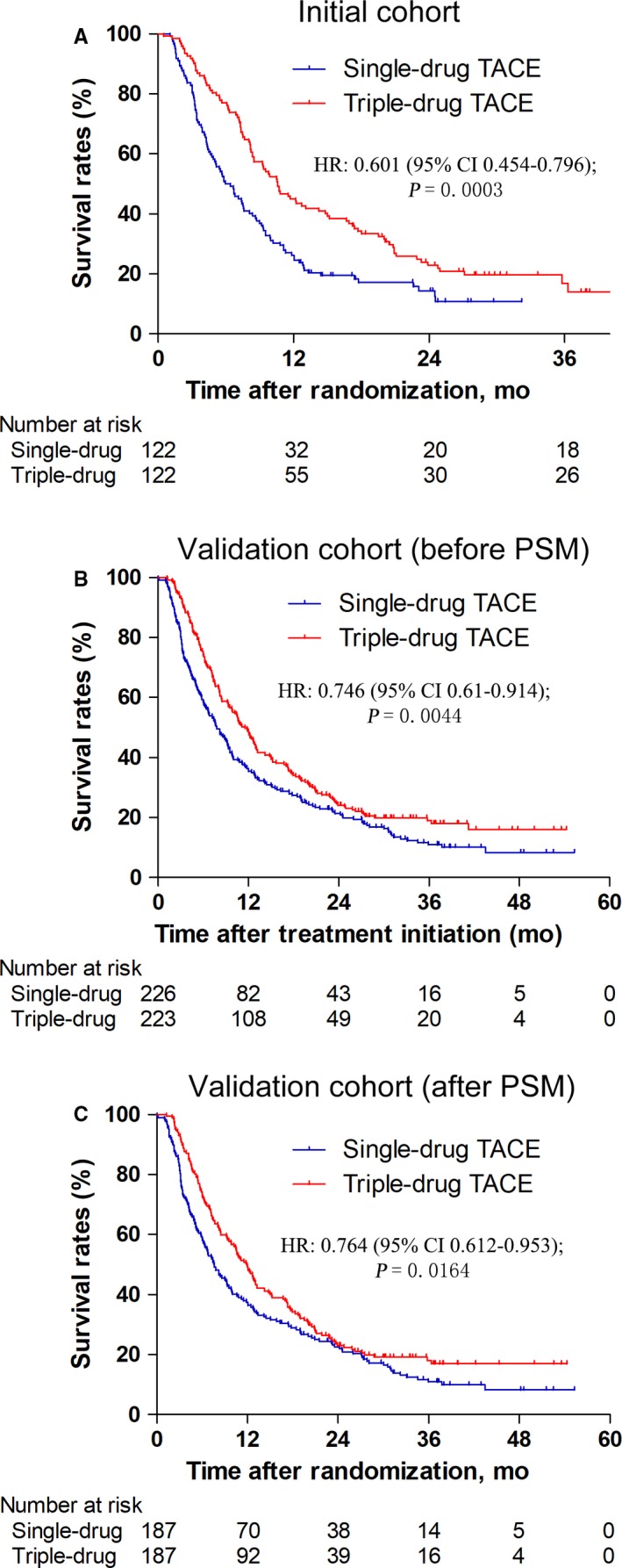
Kaplan‐Meier plots of overall survival for TACE with triple‐drug chemotherapy versus TACE with single‐drug chemotherapy. (A) Initial cohort; (B) External validation cohort (before PSM); (C) External validation cohort (after PSM). TACE, transarterial chemoembolization; PSM, propensity score matching; HR, hazard ratio

Because BCLC stage was directly related to the presence or absence of PVTT, the multivariate analysis used Model 1 (Table 2, excluding presence or absence of PVTT) and Model 2 (Table S1, excluding BCLC stage). Table 2 showed the results of univariate analysis, and Table S2 showed the survival rate (%) depending on the age, tumor size, and TACE type separately. In the initial cohort, survival rates were statistically significantly better in the triple arm (median OS = 10.567 months; 95% CI = 8.325‐12.809) than in the single arm (median OS = 5.967 months; 95% CI = 4.266‐7.668) (*P* = 0.0003) (Figure 1A). In the validation cohort (before PSM) and validation cohort (after PSM), survival rates were also statistically significantly better in the triple arm than in the single arm (11.533 [95% CI, 9.731‐13.336] vs 7.767 [95% CI, 6.232‐9.301] months, *P* = 0.0044, Figure 1B; 11.9 [95% CI, 10.278‐13.522] vs 7.567 [95% CI, 5.897‐9.236] months, *P* = 0.0164, Figure 1C).

**Table 2:** Univariate analysis and multivariate analysis for all recruited patients (model 1)

	Initial cohort			External validation cohort			External validation cohort‐PSM		
	UVA	MVA		UVA	MVA		UVA	MVA	
	*P*1	HR	*P*2	*P*1	HR	*P*1	*P*1	HR	*P*1
Group(single‐drug/triple‐drug)	0.0003	0.579	0.003	0.0044	0.714	0.0015	0.0164	0.718	0.0041
Age (≤50/>50)	0.0625	0.863	0.3525	0.0120	0.806	0.0572	0.0111	0.804	0.0823
Sex (male/female)	0.3248	0.624	0.1065	0.1048	0.745	0.1641	0.0191	0.597	0.0498
NLR (≤3/>3)	0.0119	1.288	0.1126	0.0001	1.335	0.0104	0.0081	1.192	0.1589
PT, s (≤14/>14)	0.0440	1.229	0.3161	0.0110	1.035	0.8078	0.0470	1.056	0.7375
HBsAg (positive/negative)	0.3947	1.213	0.5427	0.5189	0.812	0.2103	0.6924	0.792	0.2520
ALT,U/L (≤40/>40)	0.5694	0.752	0.1100	0.0302	1.049	0.7229	0.0308	1.058	0.7144
AST,U/L (≤45/>45)	0.0788	0.946	0.8178	0.0006	1.116	0.5336	0.0043	1.136	0.5160
ALP,U/L (≤110/>110)	0.1034	0.841	0.3078	0.0291	0.771	0.0388	0.0402	0.805	0.1083
GGT,U/L (≤100/>100)	<0.0001	2.082	0.0009	<0.0001	1.406	0.0207	<0.0001	1.342	0.0643
ALB,g/L (≤37/>37)	0.0343	1.129	0.5047	<0.0001	0.509	<0.0001	<0.0001	0.483	<0.0001
TBil, µmol/L (≤20/>20)	0.0277	1.363	0.0889	0.0013	1.072	0.5892	0.1081	0.937	0.6693
AFP,ng/ml (≤200/>200)	0.0013	1.334	0.1003	<0.0001	1.457	0.0013	<0.0001	1.509	0.0013
Tumor size,cm (≤10/>10)	0.0226	1.091	0.5925	0.0055	1.136	0.2824	0.0090	1.126	0.3524
Tumor number (single/multiple)	0.8727	1.240	0.1544	0.3679	1.165	0.1590	0.4642	1.168	0.1885
PVTT (no/yes)	<0.0001			<0.0001			<0.0001		
BCLC stage (B/C)	<0.0001	2.199	<0.001	<0.0001	1.778	<0.0001	<0.0001	1.690	<0.0001

Notes

*P*1 value was calculated with two‐sided log‐rank test. Any factors irrespective of *P* value in the univariate analysis entry into a multivariable Cox analysis.

*P*2 value was calculated by multivariable Cox proportional hazards analysis (Method: Enter).

Abbreviations: AFP, alpha‐fetoprotein; ALB, albumin; ALP, alkaline phosphatase; ALT, alanine aminotransferase; AST, aspartate aminotransferase; BCLC, Barcelona Clinic Liver Cancer; GGT, glutamyl transpeptidase; HBsAg, hepatitis B surface antigen; HR, hazard ratio; MVA, multivariate analysis; NLR, neutrophil:lymphocyte ratio; PSM, propensity score matching; PT, prothrombin time; PVTT, portal vein tumor thrombus; TBil, total bilirubin; UVA, univariate analysis.

Multivariate analysis using Model 1 (Table 2, excluding presence or absence of PVTT) showed that prognostic indicators for OS of the initial cohort, validation cohort (before PSM) and validation cohort (after PSM) were treatment allocation, GGT and BCLC stage; treatment allocation, NLR, ALP, GGT, ALB, AFP, and BCLC stage; treatment allocation, sex, ALB, AFP, and BCLC stage, respectively. The multivariate analysis using Model 2 (Table S1, excluding BCLC stage) showed that prognostic indicators for OS of the initial cohort, validation cohort (before PSM) and validation cohort (after PSM) were treatment allocation, GGT, and the presence or absence of PVTT; treatment allocation, NLR, ALP, GGT, ALB, AFP, and the presence or absence of PVTT; treatment allocation, sex, ALB, AFP, and the presence or absence of PVTT, respectively.

### 3.3 Predictive factors of treatment benefit

**Figure 2:**
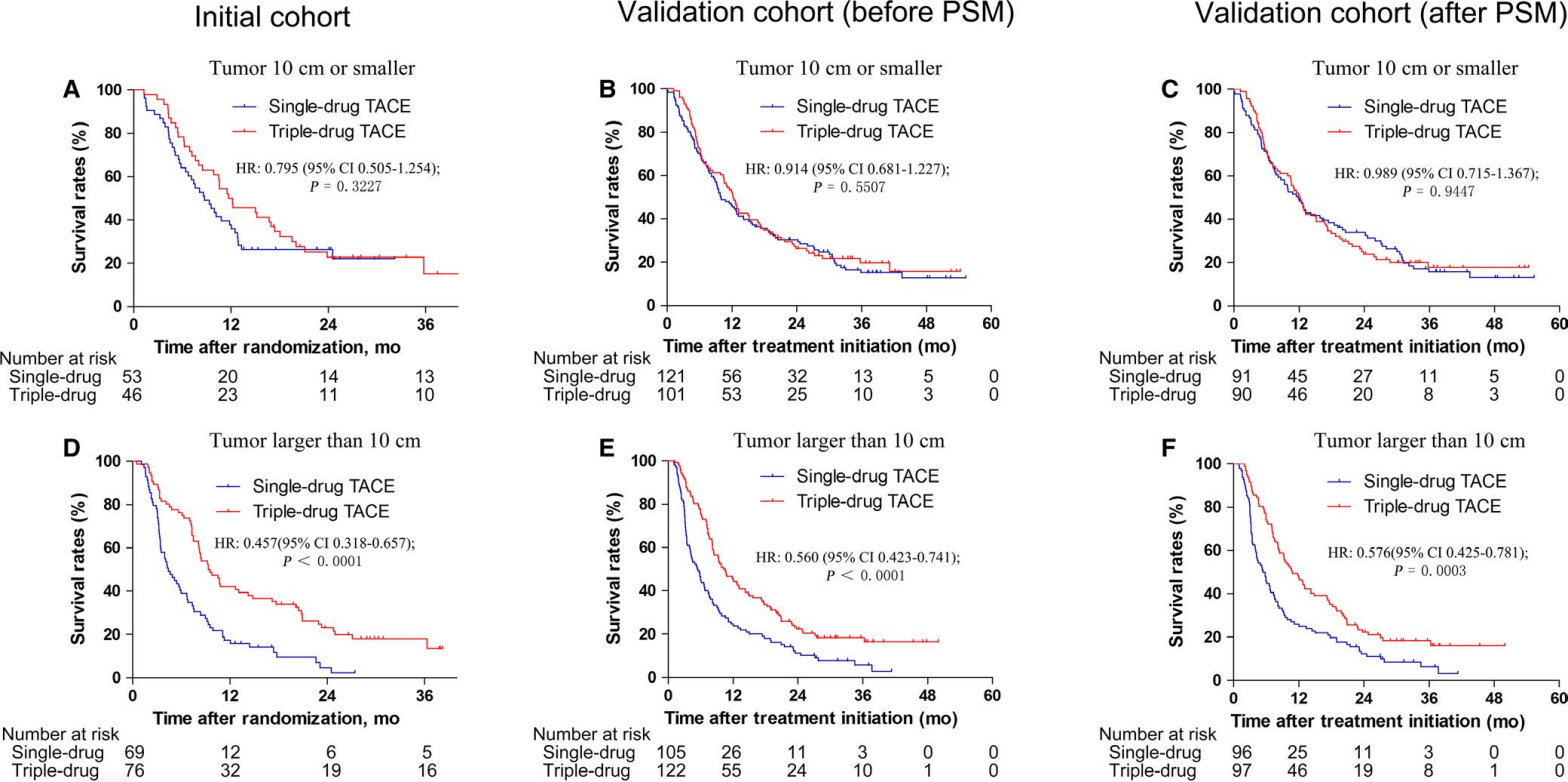
Kaplan‐Meier plots of overall survival by tumor size status for TACE with triple‐drug chemotherapy versus TACE with single‐drug chemotherapy in the initial cohort, external validation cohort (before PSM), and external validation cohort (after PSM). (A) Tumor size ≤10 cm for the initial cohort; (B) Tumor size ≤10 cm for the external validation cohort (before PSM); (C) Tumor size ≤10 cm for the external validation cohort (after PSM); (D) Tumor size >10 cm for the initial cohort; (E) Tumor size >10 cm for the external validation cohort (before PSM); (F) Tumor size >10 cm for the external validation cohort (after PSM). TACE, transarterial chemoembolization; PSM, propensity score matching; HR, hazard ratio

Predictive factors of survival benefit from triple‐drug chemotherapy in the initial cohort, validation cohort (before PSM), and validation cohort (after PSM) were summarized in Tables 3, 4, 5, respectively. Tumor size was predictive factor for the benefit of TACE with triple‐drug chemotherapy in all cohorts (interaction *P* = 0.0452, 0.0159, and 0.0153, respectively). After stratification by tumor size, there was no survival difference between the triple‐drug TACE and the single‐drug TACE in the three cohorts for tumor 10 cm or smaller (Figure 2A‐C). However, significantly greater triple‐drug chemotherapy benefit was observed in patients with tumors larger than 10 cm in the largest diameter in the initial cohort (*P* < 0.0001, Figure 2D). The survival rates were also particularly better in the triple arm than in the single arm for patients with tumors larger than 10 cm in the validation cohort (before PSM) (*P* < 0.0001, Figure 2E) and validation cohort (after PSM) (*P* = 0.0003, Figure 2F).

**Table 3:** Test for predictive value and benefit of triple‐drug TACE (initial cohort, triple arm vs single arm)

Baseline covariate	n (triple; single)	Events	Median overall survival, months		HR (95% CI) (triple/single)	Test for predictive value (Interaction) *P* (Cox Model)
			Triple arm	Single arm		
Age, y						0.9830
≤50	149 (77; 72)	127	8.467 (6.142‐10.792)	5.967 (4.304‐7.63)	0.605 (0.424‐0.862)	
>50	95 (45; 50)	74	15 (8.626‐21.374)	5.8 (1.411‐10.189)	0.581 (0.365‐0.925)	
Sex						0.47313
Men	224 (109; 115)	187	10.567 (8.654‐12.479)	5.8 (4.312‐7.288)	0.597 (0.446‐0.799)	
Women	20 (13; 7)	14	N	11.133 (4.518‐17.749)	0.399 (0.11‐1.442)	
NLR						0.9418
≤3	142 (72; 70)	116	14.233 (9.404‐19.063)	7.6 (5.174‐10.026)	0.546 (0.376‐0.794)	
>3	102 (50; 52)	85	7.467 (6.504‐8.429)	4.4 (3.559‐5.241)	0.644 (0.42‐0.988)	
PT, s						0.6821
≤14	209 (109; 100)	168	10.533 (7.836‐13.231)	6.4 (4.669‐8.131)	0.63 (0.463‐0.856)	
>14	33 (12; 21)	31	10.567 (4.909‐16.225)	4.5 (2.606‐6.394)	0.527 (0.248‐1.117)	
HBsAg						0.4913
Yes	228 (114; 114)	189	10.567 (8.503‐12.63)	5.833 (4.409‐7.258)	0.584 (0.437‐0.781)	
No	16 (8; 8)	12	9.767 (2.329‐17.204)	8.6 (0.1‐17.1)	0.886 (0.285‐2.757)	
ALT, U/L						0.2704
≤40	81 (40; 41)	66	8.467 (4.903‐12.031)	5.7 (4.111‐7.289)	0.767 (0.472‐1.244)	
>40	163 (82; 81)	135	11.667 (6.923‐16.41)	6.7 (4.512‐8.888)	0.528 (0.374‐0.747)	
AST, U/L						0.2937
≤45	47 (24; 23)	35	17.4 (9.158‐25.642)	6.4 (4.365‐8.435)	0.429 (0.214‐0.861)	
>45	197 (98; 99)	166	9.533 (7.593‐11.474)	5.967 (3.987‐7.946)	0.652 (0.479‐0.887)	
ALP, U/L						0.9173
≤110	92 (49; 43)	73	12.233 (7.793‐16.674)	7.567 (3.712‐11.421)	0.598 (0.376‐0.949)	
>110	152 (73; 79)	128	9.233 (7.224‐11.243)	5.5 (3.532‐7.468)	0.605 (0.424‐0.863)	
GGT, U/L						0.8453
≤100	60 (35; 25)	41	20.833 (15.858‐25.809)	11.267 (3.27‐19.264)	0.546 (0.291‐1.024)	
>100	184 (87; 97)	160	8.233 (7.387‐9.08)	5.433 (4.059‐6.808)	0.663 (0.484‐0.908)	
ALB, g/L						0.5588
≤37	62 (27; 35)	54	8.033 (6.281‐9.786)	5.433 (3.811‐7.056)	0.716 (0.415‐1.236)	
>37	182 (95; 87)	147	10.8 (7.536‐14.064)	6.7 (5.007‐8.393)	0.585 (0.421‐0.812)	
TBil, µmol/L						0.2550
≤20	190 (97; 93)	153	10.733 (8.16‐13.307)	6.733 (5.13‐8.337)	0.649 (0.471‐0.895)	
>20	54 (25; 29)	48	7.633 (5.729‐9.537)	4.4 (2.232‐6.568)	0.436 (0.233‐0.816)	
AFP, ng/mL						0.1935
≤200	84 (43; 41)	65	16.967 (13.754‐20.179)	9.9 (7.433‐12.367)	0.702 (0.428‐1.149)	
>200	160 (79; 81)	142	8.467 (6.823‐10.111)	4.7 (3.459‐5.941)	0.551 (0.391‐0.777)	
Tumor size, cm						0.0452
≤10	99 (46; 53)	76	11.667 (7.362‐15.971)	8.733 (6.118‐11.349)	0.795 (0.505‐1.254)	
>10	145 (76; 69)	125	9.333 (7.087‐11.58)	4.4 (2.966‐5.834)	0.457 (0.318‐0.657)	
Tumor number						0.5813
Single	102 (49; 53)	83	9.867 (8.175‐11.558)	5.967 (2.91‐9.024)	0.676 (0.439‐1.042)	
Multiple	142 (73; 69)	118	10.733 (6.702‐14.764)	6.4 (4.811‐7.989)	0.543 (0.373‐0.79)	
PVTT						0.2902
No	159 (82; 77)	120	13.067 (7.686‐18.447)	8.367 (6.016‐10.717)	0.654 (0.455‐0.94)	
Yes	85 (40; 45)	81	7.267 (6.285‐8.248)	4.267 (3.171‐5.362)	0.503 (0.32‐0.792)	
BCLC stage						0.2902
B	159 (82; 77)	120	13.067 (7.686‐18.447)	8.367 (6.016‐10.717)	0.654 (0.455‐0.94)	
C	85 (40; 45)	81	7.267 (6.285‐8.248)	4.267 (3.171‐5.362)	0.503 (0.32‐0.792)	

Note

*P* (Cox Model) was tested in the pooled treatment arms in a model containing only the baseline factor, the treatment, and their interaction.

Abbreviations: AFP, alpha‐fetoprotein; ALB, albumin; ALP, alkaline phosphatase; ALT, alanine aminotransferase; AST, aspartate aminotransferase; BCLC, Barcelona Clinic Liver Cancer; CI, confidence interval; GGT, glutamyl transpeptidase; HBsAg, hepatitis B surface antigen; HR, hazard ratio; NLR, neutrophil:lymphocyte ratio; PT, prothrombin time; PVTT, portal vein tumor thrombus; Single arm, TACE with single‐drug chemotherapy; TACE, transarterial chemoembolization; TBil, total bilirubin; Triple arm, TACE with triple‐drug chemotherapy

**Table 4:** Test for Predictive Value and Benefit of Triple‐drug TACE (external validation cohort before PSM, triple arm vs single arm)

Baseline covariate	n (triple; single)	Events	Median overall survival, months		HR (95% CI) (triple/single)	Test for predictive value (Interaction) *P* (Cox Model)
			Triple arm	Single arm		
Age, y						0.2558
≤50	240 (129; 111)	207	8.2 (6.142‐10.792)	6.8 (5.265‐8.335)	0.822 (0.626‐1.08)	
>50	209 (94; 115)	170	14.8 (10.366‐19.234)	9.1 (7.295‐10.905)	0.649 (0.479‐0.881)	
Sex						0.1217
Men	411 (210; 201)	351	10.8 (9.122‐12.478)	7.767 (6.245‐9.288)	0.769 (0.624‐0.948)	
Women	38 (13; 25)	26	N	9.1 (3.334‐14.866)	0.392 (0.156‐0.982)	
NLR						0.9326
≤3	262 (136; 126)	216	13.033 (10.312‐15.754)	9.533 (7.517‐11.55)	0.774 (0.593‐1.011)	
>3	187 (87; 100)	161	7.5 (6.484‐8.516)	4.9 (3.463‐6.337)	0.711 (0.521‐0.97)	
PT, s						0.8338
≤14	377 (195; 182)	311	11.967 (10.059‐13.874)	8.167 (6.719‐9.615)	0.765 (0.613‐0.956)	
>14	72 (28; 44)	66	10.433 (5.939‐14.928)	6.233 (3.344‐9.122)	0.735 (0.446‐1.211)	
HBsAg						0.1810
Yes	397 (201; 196)	331	10.733 (8.704‐12.762)	7.5 (6.059‐8.941)	0.783 (0.631‐0.971)	
No	52 (22; 30)	48	13.033 (7.326‐18.741)	9.1 (7.758‐10.442)	0.461 (0.249‐0.852)	
ALT, U/L						0.9342
≤40	159 (81; 78)	127	12.233 (9.98‐14.487)	8.6 (5.679‐11.521)	0.754 (0.532‐1.068)	
>40	290 (142; 148)	250	10.567 (7.647‐13.486)	7.5 (5.513‐9.487)	0.748 (0.584‐0.96)	
AST, U/L						0.1814
≤45	82 (45; 39)	62	14.233 (8.493‐19.973)	14.067 (5.501‐22.632)	1.02 (0.618‐1.684)	
>45	365 (178; 187)	315	10.567 (8.617‐12.516)	7.367 (5.96‐8.774)	0.702 (0.563‐0.877)	
ALP, U/L						0.2749
≤110	183 (100; 83)	148	12.233 (9.783‐14.683)	9.333 (5.501‐13.166)	0.862 (0.624‐1.191)	
>110	266 (123; 143)	229	10.567 (8.367‐12.766)	6.733 (5.197‐8.27)	0.691 (0.531‐0.898)	
GGT, U/L						0.8984
≤100	117 (63; 54)	89	17.933 (11.452‐24.415)	12.93 (9.689‐16.171)	0.762 (0.503‐1.155)	
>100	332 (160; 172)	288	8.467 (6.556‐10.378)	6.6 (4.886‐8.314)	0.755 (0.599‐0.952)	
ALB, g/L						0.1817
≤37	120 (52; 68)	110	8.033 (5.638‐10.428)	5.1 (3.416‐6.784)	0.612 (0.418‐0.897)	
>37	329 (171; 158)	267	12.7 (9.912‐15.488)	9.8 (7.67‐11.93)	0.821 (0.646‐1.044)	
TBil, µmol/L						0.4659
≤20	350 (179; 171)	249	12.233 (10.508‐13.959)	8.6 (7.004‐10.196)	0.783 (0.622‐0.987)	
>20	99 (44; 55)	88	7.2 (5.755‐8.645)	5.833 (2.027‐9.64)	0.665 (0.434‐1.02)	
AFP, ng/mL						0.2981
≤200	170 (79; 91)	134	16.7 (12.198‐21.202)	12.8 (8.862‐16.738)	0.85 (0.59‐1.224)	
>200	279 (144; 135)	243	8.467 (6.83‐10.104)	5.8 (4.434‐7.166)	0.702 (0.531‐0.928)	
Tumor size, cm						0.0159
≤10	222 (101; 121)	180	12.233 (10.473‐13.994)	9.933 (7.252‐12.614)	0.914 (0.681‐1.227)	
>10	227 (122; 105)	197	10.167 (7.976‐12.357)	5.433 (3.881‐6.985)	0.56 (0.423‐0.741)	
Tumor number						0.9347
Single	189 (96; 93)	158	10.567 (7.686‐13.447)	8.167 (5.561‐10.733)	0.739 (0.541‐1.011)	
Multiple	260 (127; 133)	219	11.967 (10.069‐13.864)	7.667 (5.884‐9.45)	0.753 (0.577‐0.982)	
PVTT						0.7093
No	303 (151; 152)	240	14.967 (11.661‐18.272)	9.533 (7.167‐11.899)	0.752 (0.583‐0.969)	
Yes	146 (72; 74)	137	6.367 (4.842‐7.891)	4.267 (3.108‐5.426)	0.706 (0.504‐0.987)	
BCLC stage						0.3281
B	292 (145; 147)	232	14.967 (11.574‐18.359)	9.8 (7.476‐12.124)	0.783 (0.605‐1.013)	
C	157 (78; 79)	145	7.167 (5.725‐8.609)	4.4 (3.239‐5.561)	0.66 (0.476‐0.915)	

Note

*P* (Cox Model) was tested in the pooled treatment arms in a model containing only the baseline factor, the treatment, and their interaction.

Abbreviations: AFP, alpha‐fetoprotein; ALB, albumin; ALP, alkaline phosphatase; ALT, alanine aminotransferase; AST, aspartate aminotransferase; BCLC, Barcelona Clinic Liver Cancer; CI, confidence interval; GGT, glutamyl transpeptidase; HBsAg, hepatitis B surface antigen; HR, hazard ratio; NLR, neutrophil:lymphocyte ratio; PSM, propensity score matching; PT, prothrombin time; PVTT, portal vein tumor thrombus; Single arm, TACE with single‐drug chemotherapy; TACE, transarterial chemoembolization; TBil, total bilirubin; Triple arm, TACE with triple‐drug chemotherapy.

**Table 5:** Test for predictive value and benefit of triple‐drug TACE (external validation cohort after PSM, triple arm vs single arm)

Baseline covariate	n (triple; single)	Events	Median overall survival, months		HR (95% CI) (triple/single)	Test for predictive value (Interaction) *P* (Cox Model)
			Triple arm	Single arm		
Age, y						0.2613
≤50	205 (104; 101)	178	8.2 (5.663‐10.737)	6.8 (5.268‐8.332)	0.862 (0.642‐1.158)	
>50	169 (83; 86)	137	15 (9.989‐20.011)	8.967 (5.877‐12.057)	0.673 (0.48‐0.942)	
Sex						0.2976
Men	346 (174; 172)	298	11.133 (9.362‐12.905)	7.5 (5.858‐9.142)	0.786 (0.626‐0.986)	
Women	28 (13; 15)	17	N	9.933 (2.822‐17.045)	0.467 (0.171‐1.271)	
NLR						0.7098
≤3	226 (117; 109)	190	13.033 (10.642‐15.425)	9.533 (6.774‐12.293)	0.756 (0.568‐1.005)	
>3	148 (70; 78)	125	7.467 (5.314‐9.619)	5.133 (3.438‐6.828)	0.764 (0.537‐1.087)	
PT, s						0.9852
≤14	321 (160; 161)	267	12.067 (10.156‐13.978)	8.167 (6.119‐10.215)	0.76 (0.598‐0.967)	
>14	53 (27; 26)	48	10.433 (4.949‐15.918)	4.9 (1.569‐8.231)	0.762 (0.43‐1.35)	
HBsAg						0.5862
Yes	339 (172; 167)	284	11.8 (10.134‐13.466)	7.4 (5.985‐8.815)	0.775 (0.614‐0.978)	
No	35 (15; 20)	31	12.233 (8.152‐16.315)	9.833 (5.524‐14.143)	0.614 (0.293‐1.286)	
ALT, U/L						0.9794
≤40	131 (70; 61)	104	12.233 (9.956‐14.511)	7.767 (3.211‐12.322)	0.761 (0.517‐1.118)	
>40	243 (117; 126)	211	10.967 (8.167‐13.766)	7.4 (5.698‐9.102)	0.778 (0.593‐1.02)	
AST, U/L						0.2021
≤45	70 (38; 32)	52	13.233 (6.085‐20.382)	12.8 (3.098‐22.502)	1.055 (0.61‐1.827)	
>45	304 (122; 141)	263	10.967 (8.707‐13.226)	6.733 (5.442‐8.025)	0.716 (0.562‐0.913)	
ALP, U/L						0.1124
≤110	160 (85; 75)	129	12.233 (9.523‐14.944)	11.167 (6.861‐15.473)	0.94 (0.665‐1.329)	
>110	214 (102; 112)	186	10.967 (8.135‐13.798)	6.6 (4.871‐8.329)	0.658 (0.493‐0.88)	
GGT, U/L						0.4922
≤100	97 (52; 45)	74	17.3 (11.725‐22.875)	14.167 (6.718‐21.615)	0.877 (0.556‐1.385)	
>100	277 (135; 142)	241	9.533 (7.509‐11.558)	5.967 (4.41‐7.524)	0.736 (0.571‐0.948)	
ALB, g/L						0.0876
≤37	91 (45; 46)	83	8.067 (5.306‐10.827)	4.267 (2.199‐6.335)	0.538 (0.348‐0.832)	
>37	283 (142; 141)	232	12.7 (9.78‐15.62)	9.9 (7.081‐12.719)	0.824 (0.637‐1.066)	
TBil, µmol/L						0.7449
≤20	304 (152; 152)	255	12.233 (10.471‐13.995)	8.167 (6.203‐10.13)	0.775 (0.606‐0.992)	
>20	70 (35; 35)	60	7.633 (5.246‐10.021)	5.833 (2.163‐9.504)	0.702 (0.422‐1.167)	
AFP, ng/mL						0.4511
≤200	148 (73; 75)	116	16.967 (12.439‐21.494)	13.367 (7.803‐18.93)	0.85 (0.59‐1.224)	
>200	193 (114; 112)	199	8.467 (6.428‐10.506)	5.8 (4.676‐6.924)	0.702 (0.531‐0.928)	
Tumor size, cm						0.0153
≤10	161 (90; 91)	147	12.067 (9.866‐14.267)	11.833 (8.777‐14.89)	0.989 (0.715‐1.367)	
>10	193 (97; 96)	168	10.8 (7.788‐13.812)	5.433 (3.833 1‐7.034)	0.576 (0.425‐0.781)	
Tumor number						0.9580
Single	161 (79; 82)	135	10.533 (8.727‐14.34)	7.767 (5.075‐10.459)	0.76 (0.541‐1.066)	
Multiple	213 (108; 105)	180	11.967 (10.071‐13.862)	7.367 (5.541‐9.192)	0.767 (0.573‐1.028)	
PVTT						0.4710
No	248 (125; 123)	198	14.8 (11.148‐18.452)	11.167 (7.393‐14.954)	0.795 (0.602‐1.051)	
Yes	126 (62; 64)	117	6.3 (4.985‐7.615)	4.267 (3.189‐5.345)	0.689 (0.479‐0.992)	
BCLC stage						0.1712
B	237 (119; 118)	190	14.8 (11.237‐18.363)	11.167 (6.938‐15.393)	0.84 (0.632‐1.118)	
C	137 (68; 69)	125	7.167 (5.349‐8.984)	4.4 (3.276‐5.524)	0.641 (0.45‐0.912)	

Note

*P* (Cox Model) was tested in the pooled treatment arms in a model containing only the baseline factor, the treatment, and their interaction.

Abbreviation: AFP, alpha‐fetoprotein; ALB, albumin; ALP, alkaline phosphatase; ALT, alanine aminotransferase; AST, aspartate aminotransferase; BCLC, Barcelona Clinic Liver Cancer; CI, confidence interval; GGT, glutamyl transpeptidase; HBsAg, hepatitis B surface antigen; HR, hazard ratio; NLR, neutrophil:lymphocyte ratio; PSM, propensity score matching; PT, prothrombin time; PVTT, portal vein tumor thrombus; Single arm, TACE with single‐drug chemotherapy; TACE, transarterial chemoembolization; TBil, total bilirubin; Triple arm, TACE with triple‐drug chemotherapy.

### 3.4 Safety

Our previous showed that no difference in the incidence of adverse event or serious adverse event was observed between the triple arm and single arm.^15^ For the validation cohort, there was no difference in any grade or grade 3‐4 adverse event between the two arms (Table S3).

## 4 DISCUSSION

This study showed that triple‐drug TACE significantly prolonged the survival of patients with unresectable HCC in all cohorts, which was consistent with our previous phase III study.^15^ It may be explained that chemotherapy played a positive and major effect in TACE, and our another study showed that hepatic artery infusion chemotherapy (abandoning embolization completely and strengthening chemotherapy) had better tumor responses and less serious adverse events than TACE.^9^ Then, the triple‐drug TACE were common in clinical used in our hospital,^9^, ^22^ and the overall efficacy of triple‐drug TACE in patients with intermediate HCC seen in another study^22^ was comparable with that reported in our previous phase III study^15^ (17.67 vs 13 months). Treatment allocation and BCLC stage (or PVTT) were independent negative prognostic factors in all cohorts. There were discrepancies of identified prognostic factors between this study and original study.^15^ That might be explained: First, sample size varied greatly; Second, initial and validation cohort were from different hospital; Finally, the multivariate analysis of original study included three arms (triple‐drug TACE with embolization arm, single‐drug TACE arm with embolization, and triple‐drug TACE without embolization arm) while this study only included two arms (triple‐drug TACE with embolization arm and single‐drug TACE arm with embolization).

The purpose of this study was to find out particular patients who gained the greatest benefit from triple‐drug chemotherapy. In the initial cohorts, validation cohort (before PSM), and validation cohort (after PSM), tumor size was the strongest baseline predictive factor for the benefit of triple‐drug chemotherapy. A significant survival difference was seen in large tumors (>10 cm) but no survival difference was seen in small tumors (≤10 cm) between triple‐drug chemotherapy and single‐drug chemotherapy. Several explanations could account for this result. First, small HCCs had fewer feeding arteries and were less invasive; thus, it is feasible to completely block all tumor‐feeding arteries. However, it is especially difficult for large HCCs to completely block all tumor‐feeding arteries because these HCCs usually have multiple intrahepatic or extrahepatic collateral circulation.^23^, ^24^ Second, embolization could only block the hepatic artery. However, as tumors grow, more HCCs accompanied hepatic arteriovenous shunts,^25^ and the hepatic artery supply decreased while the portal vein supply increased. Third, large tumors needed more embolic agents while the amount of embolic agents was limited because patients with large tumors frequently had poor liver reserve.^23^, ^26^ Finally, chemotherapy drugs can flow to the whole liver by means of a hepatic artery portal vein traffic branch that was not affected by the tumor blood supply vessels. Tumor size was also one of the strongest prognostic factors for survival in HCC,^27^ and previous studies showed that TACE for large HCC had a poor prognosis.^23^, ^28^ Guidelines from the American Association for the Study of Liver Diseases showed that tumor burden (size >10 cm) is relative to contraindications for TACE.^29^ Our previous studies also showed that TACE without embolization did not reduce survival,^15^ and hepatic artery infusion chemotherapy had better tumor responses and less serious adverse events than TACE.^9^ Therefore, embolization might be ineffective, and chemotherapy might play the major role in large HCCs. Whether embolization plays a major role in small HCCs requires further research.

Our findings could explain why different studies investigating TACE with combination chemotherapy vs TACE with mono‐chemotherapy had contrasting results. One study with large tumors (mean size = 8 cm) showed that TACE with combination chemotherapy had better survival than did TACE with mono‐chemotherapy,^30^ while another study with small tumors (mean size ≤3 cm) showed no survival difference.^31^ In addition, the reason why studies of TACE vs transarterial embolization without chemotherapy^32^, ^33^, ^34^, ^35^, ^36^, ^37^ showed no survival difference could be explained. First, most studies had small tumors (mean size ≤5 cm), except for the study of Malagari K (mean size >8 cm).^37^ Second, all studies used a single chemotherapeutic drug. Doxorubicin or cisplatin alone might be ineffective. Whether a single chemotherapeutic drug is sufficient to cause a survival difference requires further study. Third, different chemotherapeutic drugs might cause a survival difference. Compared with cisplatin, lobaplatin was reported to have less toxicity, better therapeutic index, and higher solubility.^38^, ^39^

There were many limitations in this study. The first limitation of this trial was the retrospective nature of the external validation cohort; however, there were no significant differences in the baseline characteristics between the two arms, and the same results were attained after PSM was used. Because this was not a prespecified hypotheses but a post hoc exploratory analysis, this result needs confirmation in other prospective trials. The second limitation was that TACE was not the current standard treatment for HCC with PVTT. However, previous studies showed that TACE was the safe and effective treatment for HCC patients with PVTT.^40^, ^41^ Finally, this study was done only in China. The predominant etiology of HCC in China was hepatitis B virus. Therefore, whether the results could be adapted to western countries where the etiology of HCC is mainly hepatitis C virus remains to be proved.

In summary, this retrospective explorative trial showed that TACE with triple‐drug chemotherapy had better survival rates than TACE with single‐drug chemotherapy, particularly in patients with large tumors (>10 cm). However, patients with small tumors (≤10 cm) could not benefit from the chemotherapy of TACE. Tumor size was a predictive factor for the benefit of TACE with triple‐drug chemotherapy.

## CONFLICT OF INTEREST

The authors do not have any disclosures to report.

## AUTHORS CONTRIBUTIONS

MinKe He, Qing Li, RuHai Zou, JingXian Shen, Ming Shi involved in conceptualization, methodology, software, validation and formal analysis, and visualization. MinKe He, JiaYing Lai, Qing Li, Ming Shi written the original draft. Ming Shi involved in supervision, project administration, and funding acquisition. All the authors involved in investigation, resources, data curation, and writing – review and editing, and final approval of manuscript.
