# Supplementary material for: Predictive factors for the benefit of triple‐drug transarterial chemoembolization for patients with unresectable hepatocellular carcinoma
Source: Cancer Med. 2019 Jun 17;8(9):4200–13. doi: 10.1002/cam4.2355 (PMC6675716; doi:10.1002/cam4.2355)
Supplement: Supplementary file 1 [file CAM4-8-4200-s001.docx]

**Supplement Tables**

**Other characteristics for Predictive Value and Benefit was shown as follows**

**Table 1s. Univariate analysis and** **multivariate analysis for all recruited patients (model 2) ***

|  | Initial cohort | | |  | External validation cohort | | |  | External validation cohort-PSM | | |
| --- | --- | --- | --- | --- | --- | --- | --- | --- | --- | --- | --- |
|  | UVA | MVA | |  | UVA | MVA | |  | UVA | MVA | |
|  | *P*1 | HR | *P*2 |  | *P*1 | HR | *P*1 |  | *P*1 | HR | *P*1 |
| Group(single-drug/triple-drug) | 0.0003 | 0.579 | 0.003 |  | 0.0044 | 0.719 | 0.0019 |  | 0.0164 | 0.725 | 0.0053 |
| Age(≤50/＞50) | 0.0625 | 0.863 | 0.3525 |  | 0.0120 | 0.816 | 0.0739 |  | 0.0111 | 0.814 | 0.1030 |
| Sex(male/female) | 0.3248 | 0.624 | 0.1065 |  | 0.1048 | 0.733 | 0.1406 |  | 0.0191 | 0.582 | 0.0393 |
| NLR(≤3/＞3) | 0.0119 | 1.288 | 0.1126 |  | 0.0001 | 1.346 | 0.0082 |  | 0.0081 | 1.204 | 0.1361 |
| PT, s(≤14/＞14) | 0.0440 | 1.229 | 0.3161 |  | 0.0110 | 1.028 | 0.8449 |  | 0.0470 | 1.044 | 0.7910 |
| HBsAg(positive/negative) | 0.3947 | 1.213 | 0.5427 |  | 0.5189 | 0.811 | 0.2090 |  | 0.6924 | 0.784 | 0.2325 |
| ALT,U/L(≤40/＞40) | 0.5694 | 0.752 | 0.1100 |  | 0.0302 | 1.023 | 0.8681 |  | 0.0308 | 1.025 | 0.8695 |
| AST,U/L(≤45/＞45) | 0.0788 | 0.946 | 0.8178 |  | 0.0006 | 1.143 | 0.4463 |  | 0.0043 | 1.176 | 0.4094 |
| ALP,U/L(≤110/＞110) | 0.1034 | 0.841 | 0.3078 |  | 0.0291 | 0.773 | 0.0412 |  | 0.0402 | 0.806 | 0.1102 |
| GGT,U/L(≤100/＞100) | ＜0.0001 | 2.082 | 0.0009 |  | ＜0.0001 | 1.376 | 0.0296 |  | ＜0.0001 | 1.305 | 0.0918 |
| ALB,g/L(≤37/＞37) | 0.0343 | 1.129 | 0.5047 |  | ＜0.0001 | 0.520 | ＜0.0001 |  | ＜0.0001 | 0.494 | ＜0.0001 |
| TBil, µmol/L(≤20/＞20) | 0.0277 | 1.363 | 0.0889 |  | 0.0013 | 1.052 | 0.6951 |  | 0.1081 | 0.914 | 0.5530 |
| AFP,ng/ml(≤200/＞200) | 0.0013 | 1.334 | 0.1003 |  | ＜0.0001 | 1.473 | 0.0010 |  | ＜0.0001 | 1.525 | 0.0010 |
| Tumor size,cm(≤10/＞10) | 0.0226 | 1.091 | 0.5925 |  | 0.0055 | 1.124 | 0.3239 |  | 0.0090 | 1.112 | 0.4033 |
| Tumor number(single/mutiple) | 0.8727 | 1.240 | 0.1544 |  | 0.3679 | 1.148 | 0.2008 |  | 0.4642 | 1.151 | 0.2328 |
| PVTT(no/yes) | ＜0.0001 | 2.199 | ＜0.001 |  | ＜0.0001 | 1.858 | ＜0.0001 |  | ＜0.0001 | 1.792 | ＜0.0001 |
| BCLC stage(B/C) | ＜0.0001 |  |  |  | ＜0.0001 |  |  |  | ＜0.0001 |  |  |

Abbreviations: UVA, univariate analysis; MVA, multivariate analysis; HR, hazard ratio; PSM, propensity score matching; NLR, neutrophil:lymphocyte ratio; PT, prothrombin time; HBsAg, hepatitis B surface antigen; ALT, alanine aminotransferase; AST, aspartate aminotransferase; ALP, alkaline phosphatase; GGT, glutamyl transpeptidase; ALB, albumin; TBil, total bilirubin; AFP, alpha-fetoprotein; PVTT, portal vein tumor thrombus; BCLC, Barcelona Clinic Liver Cancer.

*P*1 value was calculated with two-sided log-rank test. Any factors irrespective of *P* value in the univariate analysis entry into a multivariable Cox analysis.

*P*2 value was calculated by multivariable Cox proportional-hazards analysis (Method: Enter).

**Table 2s. Survival rate (%) depending on the age, tumor size, and TACE type separately**

|  | initial cohort | | | |  | validation cohort (before PSM) | | | |  | validation cohort (after PSM) | | | |
| --- | --- | --- | --- | --- | --- | --- | --- | --- | --- | --- | --- | --- | --- | --- |
|  | 3 mon | 6 mon | 9 mon | 12 mon |  | 3 mon | 6 mon | 9 mon | 12 mon |  | 3 mon | 6 mon | 9 mon | 12 mon |
| Age, year |  |  |  |  |  |  |  |  |  |  |  |  |  |  |
| ≤50 | 86.58% | 60.40% | 40.94% | 28.86% |  | 86.25% | 61.25% | 44.17% | 35.83% |  | 86.83% | 60.49% | 44.39% | 36.59% |
| ＞50 | 88.42% | 68.42% | 56.84% | 46.32% |  | 88.52% | 71.77% | 61.24% | 49.76% |  | 89.35% | 71.60% | 62.13% | 51.48% |
| Tumor size, cm |  |  |  |  |  |  |  |  |  |  |  |  |  |  |
| ≤10 | 91.92% | 70.71% | 55.56% | 43.43% |  | 89.64% | 71.17% | 59.01% | 49.10% |  | 90.61% | 71.27% | 59.67% | 50.28% |
| ＞10 | 84.14% | 58.62% | 41.38% | 30.34% |  | 85.02% | 61.23% | 45.37% | 35.68% |  | 85.49% | 60.10% | 45.60% | 36.79% |
| TACE type |  |  |  |  |  |  |  |  |  |  |  |  |  |  |
| Single-drug | 82.79% | 50.00% | 36.89% | 26.23% |  | 81.42% | 58.41% | 45.58% | 36.28% |  | 82.89% | 58.29% | 44.92% | 37.43% |
| Triple-drug | 91.80% | 77.05% | 57.38% | 45.08% |  | 93.27% | 73.99% | 58.74% | 48.43% |  | 93.05% | 72.73% | 59.89% | 49.20% |

Abbreviations: Triple arm, TACE with triple-drug chemotherapy; Single arm, TACE with single-drug chemotherapy; TACE, transarterial chemoembolization; PSM, propensity score matching.

**Table 3s. Incidence of TACE-Related Adverse Events for the external validation cohort before PSM.**

|  | Triple arm (n=201) | |  | Single arm (n=199) | |  | P value | |
| --- | --- | --- | --- | --- | --- | --- | --- | --- |
|  | Any grade | Grade 3-5 |  | Any grade | Grade 3-5 |  | Any grade | Grade 3-5 |
| Hematologic toxicity |  |  |  |  |  |  |  |  |
| Neutropenia | 34 | 4 |  | 28 | 2 |  | 0.4903 | 0.6852 |
| Anemia | 76 | 15 |  | 60 | 8 |  | 0.1141 | 0.1968 |
| Thrombocytopenia | 75 | 27 |  | 68 | 22 |  | 0.5326 | 0.5425 |
| Liver dysfunction |  |  |  |  |  |  |  |  |
| Elevated ALT level | 156 | 102 |  | 142 | 93 |  | 0.1691 | 0.4258 |
| Elevated AST level | 167 | 111 |  | 160 | 108 |  | 0.5191 | 0.9200 |
| Hyperbilirubinemia | 113 | 23 |  | 121 | 26 |  | 0.3628 | 0.6498 |
| Hypoalbuminemia | 142 | 1 |  | 153 | 2 |  | 0.1733 | 0.6222 |

Abbreviations: Triple arm, TACE with triple-drug chemotherapy; Single arm, TACE with single-drug chemotherapy; TACE, transarterial chemoembolization; PSM, propensity score matching; ALT, alanine aminotransferase; AST, aspartate aminotransferase.

P values were calculated using a two-sided Chi square test

22 patients in the triple arm and 27 patients in the single arm had no laboratory tests after TACE, leaving 201 patients in the triple arm and 199 patients in the single arm.
